# Mare’s Milk from a Small Polish Specialized Farm—Basic Chemical Composition, Fatty Acid Profile, and Healthy Lipid Indices

**DOI:** 10.3390/ani11061590

**Published:** 2021-05-28

**Authors:** Grażyna Czyżak-Runowska, Jacek Antoni Wójtowski, Romualda Danków, Daniel Stanisławski

**Affiliations:** 1 Department of Animal Breeding and Product Quality Assessment, Faculty of Veterinary Medicine and Animal Science, Poznan University of Life Science, Złotniki, ul. Słoneczna 1, 62-002 Suchy Las, Poland; grazyna.czyzak-runowska@up.poznan.pl; 2 Department of Dairy Products Quality, Poznań University of Life Sciences, ul. Wojska Polskiego 31, 60-624 Poznań, Poland; romualda.dankow@up.poznan.pl; 3 Computer Lab, Poznań University of Life Sciences, Wołynska 33, 60-637 Poznan, Poland; daniel.stanislawski@up.poznan.pl

**Keywords:** mare’s milk, lactation, fatty acids, birth order, atherogenic and thrombogenic indices

## Abstract

**Simple Summary:**

As mare’s milk has high nutritional and biological value, it has a number of therapeutic properties. The aim of this study was to determine the basic composition, fatty acid profile, and values of health-related indices of milk from mares of different lactation stages, ages, and birth orders. The study was conducted on milk obtained from Coldblood mares in weeks 10, 15, and 25 of lactation. The mares were aged between five and 14 years. It was found that milk produced from the 15th week of lactation had the most beneficial fatty acid composition and very beneficial values of health-related indices (low values of the atherogenic index—AI and thrombogenic index—TI), which is important with regard to the prevention of atherosclerosis and thrombosis. Moreover, the study found a dependence between birth order and the atherogenic index, which was lower in milk derived from older mares (birth order > 7). The findings from the study indicate that it is possible to modify the fatty acid profile of milk by appropriately managing the age structure of the herd of mares, among other things. To confirm this dependence, the study will be continued on a larger group of mares.

**Abstract:**

The objective of this study was to determine the chemical composition, fatty acid profile, and values of healthy indices of milk from a specialized farm of Polish Coldblood mares of different ages, birth orders, and lactation stages. Milk samples (*n* = 48) were collected for analysis in weeks 10, 15, and 25 of lactation from mares aged between five and 14 years. The study showed that the stage of lactation has a significant effect on the fatty acid (FA) profile of the milk produced on the farm. The highest concentration of monounsaturated and polyunsaturated FAs was found in milk produced from the 15th week of lactation. The milk was also characterized by low values of atherogenic and thrombogenic indices, which indicate the health benefits of milk with respect to the content of fatty acids and their potential to prevent or cause atherosclerosis and thrombosis. The study also found a significant correlation between the number of foalings (birth order), the fatty acid profile, and atherogenic index of milk produced on the farm. The findings from the study indicate that it is possible to modify the fatty acid profile of bulk tank milk through appropriate management of the age structure of the herd of mares. To confirm this dependence, the study will be continued on a larger group of mares.

## 1. Introduction

The health-promoting properties of food products are increasingly shaping the market for functional foods. This is also the case for non-cow milk and non-cow milk products, particularly equine milk [1,2]. The increased interest in equine milk is due to the unique characteristics of this type of milk and products made from it [3,4]. Mare’s milk has valuable nutritional and therapeutic properties and is recommended in the diet of people with weakened immunity including, in particular, the elderly and convalescents [5,6,7]. Mare’s milk is also believed to be useful in preventing atherosclerosis [8]. The composition of mare’s milk differs considerably from the composition of milk of major dairying species (i.e., the cow, buffalo, goat, and sheep). Mare’s milk contains three times less fat than cow’s milk. The average total protein content of mare’s milk is much lower than that of cow’s milk, but almost 50% higher than that of human milk. The content of lactose, which facilitates calcium absorption in the intestine, among other things, in mare’s milk is similar to the lactose content of breast milk and much higher than the lactose content of cow’s milk [9]. The mineral content of mare’s milk is almost twice lower compared to that of cow’s milk and is closer to the mineral content of breast milk [10]. The whey protein content of mare’s milk (0.83%) is higher than that of cow’s milk (0.57%) and that of breast milk (0.76%). As a result, mare’s milk contains higher levels of functional substances, hormones, immunoglobulins, and nitrogen compounds displaying antibacterial activity including (e.g., lysozyme and lactoferrin [11,12]). The immunoglobulin content of equine milk (mare’s and donkey’s milk) is almost twice higher than that of cow’s milk and slightly higher than the immunoglobulin content of breast milk [9]. The lactoferrin content of mare’s and donkey’s milk is significantly higher than that of cow’s milk [11,13]. However, it is three times lower than the lactoferrin content of breast milk. In turn, the lysozyme content of mare’s milk is almost four times higher than that of breast milk [9,12].

Compared with cow’s milk, equid milk, and mare’s milk in particular, has high hygienic quality and low somatic cell counts [14]. The fat components of equid milk have particular dietary importance. Equid milk, compared to cow’s milk, contains lower SFA amounts and higher essential fatty acid (EFA) amounts. This suggests that equid milk is more like human milk than cow’s milk [8,15,16,17]. Recently, there has been increasing interest in equid milk due to its potential role in human nutrition [18]. Determining the characteristics of equid milk, in particular mare’s milk, is important for economic interest in human diets and pharmaceuticals [19]. The composition of mare’s milk is influenced by genetic factors (breed) and environmental factors [20]. Knowledge of the effects of these factors will significantly facilitate the management of specialized mare’s milk farms as it will provide precise guidance on when consumer milk should be obtained in order to ensure that the process does not interfere with the development of foals and that the milk obtained has the highest possible content of health-promoting nutrients. Thus, the objective of this study was to characterize the chemical composition, fatty acid profile, and value of health-related indices of milk from a specialized farm of Polish Coldblood mares of different ages, birth orders, and lactation stages.

## 2. Materials and Methods

### 2.1. Animal and Farm Characteristics

The experiment was carried out on a small mare’s milk farm. The farm is located in northwestern Poland (Kłodzin, 52041′39″N; 17023′6″E) in the Wielkopolska region and has an area of 33 ha including 2.5 ha of grassland. It has been operating for 15 years and produces mare’s drinking milk, which is supplied frozen to customers by a courier service. The milk is placed in plastic 250 mL containers before being frozen. The farm keeps 13 Polish Coldblood, Silesian, and Haflinger mares. Milk is produced on the farm in a manner ensuring the least possible interference with nature. The foals stay with their mothers throughout the whole period of lactation. Milking begins in the third month of lactation and the mares continue to be milked for the next 3–4 months. The mares are milked once a day. During the milking period, the foals are separated from their mothers for only 3–4 h a day before milking. During that time, foals stay in the paddock, where they have access to crushed oats and barley, whereas their mothers stay in stalls. The mares are restrained in a crush during milking. Foals return to their mothers immediately before milking and can see and hear them during the milking process. After milking, the mares and their foals return to their stalls or are released into the paddock. The mares have free access to pasture, the botanical composition of which is 70% grasses (mainly perennial ryegrass), 20% legumes, and 10% others including herbs, and to water and mineral salt blocks. 

The experimental milk was collected from eight Polish Coldblood mares. The mares were from five to 14 years of age between the 10th and 25th week of lactation and with an average body weight of 618 kg. All were in a similar physical condition. Every day, the mares received 6 kg of concentrate including 4 kg of oats and 2 kg of barley, divided into two rations. While in the paddock, the horses had access to pasture forage as well as to good-quality meadow hay and oat straw. From July to October, fresh corn forage was available in the paddock. 

The rations were calculated to meet the daily nutrient requirements of lactating mares of 600 kg mature weight, according to the Polish Nutrient Requirement Standards for Horses [21], which are as follows: for mares in the 1st–3rd months of lactation—12.5 kg dry matter, 142 MJ digestible energy, 1185 g digestible crude protein, 61 g Ca, 46 g P, 70 g NaCl, and 100 mg carotene; and for mares in the 4th–6th months of lactation—11 kg dry matter, 119 MJ digestible energy, 885 g digestible crude protein, 52 g Ca, 37 g P, 51 g NaCl, and 90 mg carotene.

### 2.2. Milk Samples and Chemical Analyses

Individual milk samples (*n* = 48) from eight Polish Coldblood mares, foaled in the period May–July, were collected in the 10th, 15th, and 25th week postpartum. In each of those weeks, milk samples were collected for testing twice—on the first (Monday) and fifth (Friday) day of the week. Week 10 is the week when the mares start to be milked on the farm, week 15 is usually when the highest individual milk yield is recorded, whereas week 25 is the week when a significant decrease in milk yield is observed and is one of the last weeks when milk is obtained from the mares on the farm. Mares were always milked at the same time of the day, between 12:00 and 13:00 p.m., 3 h after separating foals from their mothers. The mares were machine milked. A milking machine for goats adapted to a mare’s udder was used, with a mean vacuum level of 45 kPa, a pulsation rate of 110 cycles/min, and a pulsation ratio of 1:1. During each milking session, mares were given small quantities of concentrate (200–300 g per milking).

The milk samples were then preserved (−30 °C) in individually coded containers until and during analysis. The fat, protein, lactose, and dry matter contents were determined using an infrared milk analyzer Milkoscan FT 120 instrument (Foss Electric, Hillerod, Denmark). Somatic cell count (SCC) was evaluated using a Bacto Count IBCm (Bentley Instruments Inc., Chaska, MN, USA) according to the ISO 21187 standard [22]. A Bacto Count apparatus was calibrated according to the ISO 4833 [23] standard (Bentley Polska Sp. o. o., Warsaw, Poland).

The fatty acid composition of the milk samples collected was determined using capillary gas chromatography. Milk fat was extracted with chloroform:methanol (2:1, *vol/vol*) using the method described by Folch et al. [24]. The fatty acid composition in the milk was estimated using methyl esters prepared by direct transesterification, according to the IUPAC method [25]. The analyses were performed using a Hewlett Packard model 6890NGC (Agilent Technologies, Palo Alto, CA, USA) equipped with a flame ionization detector, autosampler, and split/splitless injector. Separations were performed on a BPX70 column (60 m × 0.22 mm i.d. 0.25× µm film thickness, stationary phase 70% cyanopropylpolysilphenylene-siloxane; SGE Analytical Science, Austin, TX, USA). The conditions for the chromatographic analysis were as follows: the injector temperature was 230 °C with a split ratio set to 100:1 and the FID temperature was 270 °C. The oven temperature was ramped from 130 °C (3 min) to 235 °C (5 min) at a rate of 2 °C min^−1^. Helium was used as a carrier gas with a constant pressure of 40 psi at a flow rate of 0.3 mL min^−1^ and an injection volume of 1 µL. 

The fatty acid content is expressed as a percentage of the total fatty acids identified.

SFA= C4:0, C6:0, C8:0, C10:0, C12:0, C13 iso, C14:0, C15:0, C16:0, C17:0, C18:0, C20:0.
SFA (saturated fatty acids)—sum of SCFA + MCSFA + LCSFA.
SCSFA (short-chain saturated fatty acids)—sum of C4:0 + C6:0.
MCSFA (middle-chain saturated fatty acids)—sum of C8:0 + C10:0 + C12:0.
LCSFA (long-chain saturated fatty acids)—sum of C13:0 + C14:0 + C15:0 + C16:0 + C17:0 + C18:0 + C20:0.
UFA (unsaturated fatty acids)—total sum of MUFA + PUFA.
MUFA (monounsaturated fatty acids)—sum of C10:1 + C12:1 + C14:1 + C15:1 + C16:1 + C17:1 + C18:1 trans + C18:1 cis 9 + C18:1 cis 11 + C20:1.
PUFA (polyunsaturated fatty acids)—sum of C18:2 + C18:3n3 + C18:2c9t11(CLA) + C20:2 + C20:3n6 + C20:4n6 + C22:5EPA+ C20:3n3 + C22:5n3.
PUFA/SFA—ratio of polyunsaturated fatty acids and saturated fatty acids.
MUFA/SF—ratio of monounsaturated fatty acids and saturated fatty acids.
LA/ALA ratio: of C18:2n6 (linoleic acid) and C18:3n3 (α-linolenic acid): = C18:2n6/C18:3n3.
*n*-6 PUFA—sum of C18:2n6 + C20:2 + C20:3 + C20:4.
*n*-3 PUFA—sum of C18:3n3 + 20:3n3 + C20:5EPA + C22:5n3
*n*-6 PUFA/*n*-3 PUFA ratio
DFA—desirable fatty acids: (UFA + C18:0).
CLA—conjugated linoleic acid (C18:2c9t11).
AI—Atherogenic index [C12:0 + (4 × C14:0) + C16:0]/[MUFA + (*n*-6 PUFA) + (*n*-3 PUFA)].
TI—Thrombogenic index (C14:0 + C16:0 + C18:0)/(0.5 × MUFA) + (0.5 × *n*-6 PUFA + 3 × *n*-3 PUFA) + (*n*-3 PUFA/*n*-6 PUFA).

The atherogenic (AI) and thrombogenic (TI) indices were calculated using the Ulbricht and Southgate equations [26].

### 2.3. Statistical Analysis

The data collected (mare number, sample number, lactation, and lactation phase) were coded to ensure that the data analysis was not influenced during or after the trial until analyses were complete.

The data were evaluated with statistical software SAS 9.4 2018 (SAS Institute Inc., Cary, NC, USA). The MEANS and UNIVARIATE procedures were used to calculate the descriptive statistics.

The GLM procedure was used for detailed analysis of the influence of the experimental factors (lactation, phase of lactation, age of the mares) on milk composition and fatty acid profile. 

The model equation used for the evaluation of analyzed traits was as follows:

Y_ijk_ = μ + l_i_ + p_j_ + b_1_ × ma_k_ + e_ijkl_ (1)

where:

Y_ijk_—the phenotypic value of the trait analyzed (FA content);
μ—the overall mean;
l_i_—fixed effect of i-th lactation; i = 1,2; 1 ≤ 7 (n = 21); 2 > 7 (n = 27);
p_j_—fixed effect of j-th phase of lactation; j = 1,2,3; 1 = 10, 2 = 15 and 3 = 25 week;
b_1_—1st degree partial linear regression coefficient;
ma_k_—age of mare (5–10 years n = 23 and 11–14 years n = 25);
e_ijkl_—random residual effect.

A detailed comparison of object means was performed using Tukey’s test. Differences were considered significant at *p* ≤ 0.05.

The data relating somatic cell count (SCC) in the milk were subjected to logarithmic transformation (natural logarithm, LN) before statistical verification [27].

## 3. Results and Discussion

### 3.1. Basic Chemical Composition of Milk

The basic composition of the mare’s milk is presented in Table 1. The average fat content in the periods of lactation analyzed was very stable at 1.16%. It was similar to the fat content of Arabian mare’s milk (1.16%) [28] and milk produced by hand-milked Murgese mares (1.06%) [29]. The fat content found in our study was lower than the value reported by Salamon et al. [30] (1.25%), but higher than the 0.73% reported by Barreto et al. [31] for milk from Quarter Horse mares and the 0.43% observed in the late lactation of Polish Half Bred mares [32].

In our study, the highest percentage protein content and dry matter content, 2.25% and 9.65%, respectively, were found in the 10th week after foaling. The concentration of milk components, except for percentage fat content, decreased over time and was significantly correlated with the stage of lactation (*p* ≤ 0.05). In their study on the milk of Quarter Horse mares, Barreto et al. [31] noticed a similar trend and found a significant influence of the stage of lactation on protein and dry matter content. 

Compared with the milk of other mammalian species, mare’s milk has a higher lactose content [8]. In our study, lactose content was 6.44% on average and was influenced by the stage of lactation (*p* = 0.0139) and birth order (*p* = 0.0321). Lactose content was significantly higher in milk obtained from mares with seven lactations or less and decreased with the age of the animals (*p* = 0.0338). A study by Barreto et al. [31] on mare’s milk also found an effect of birth order on lactose content. The authors of the study found that lactose levels were highest during the first two lactations and decreased significantly according to the maturity of the glandular tissue. 

Studies by other authors have also found that the stage of lactation has a predominant effect on the composition of mare’s milk. In a study by Pikul et al. [33], the percentage fat content in the period from the 1st to the 5th month after foaling was 1.51%, with the highest fat content observed in the first two months of lactation (*p* ≤ 0.05).

The cytological quality of the milk samples analyzed in our study, as expressed by somatic cell count, was good, namely 16.3 × 10^3^/mL. The highest SCC was found in the 25th week of lactation (*p* ≤ 0.05), which may suggest that it was the period when lactation was gradually progressing to its end stage. In their study, Končurat et al. [34] found a similar SCC in the milk of Croatian Coldblood mares on the 60th and 120th day after foaling. The SCC at these two time points was 14 and 34 × 10^3^/mL, respectively.

### 3.2. Fatty Acid Profile of Milk

The fatty acid profile (% of total fatty acids) of the milk is presented in Table 2. Fatty acid levels were primarily influenced by the stage of lactation. In general, the levels of most saturated FAs decreased as lactation progressed and were highest in the 10th week of lactation. An inverse relationship was found for most unsaturated FAs, with the percentage of those fatty acids in total fatty acids increasing during the course of lactation. 

Means (in rows) marked with different letters differ statistically (*p* ≤ 0.05): a, b, c—for lactation stage, d, e—for number of foalings.

The effect of the number of foalings was statistically significant only in the case of eight FAs: C6:0, C8:0, C10:0, C16:1, C18:0, C18:2 *n*-6 (LA), C20:1, and C20:2. The age of the mares had a significant effect only on the following FAs: C6:0, C8:0, C10:0, C16:1, C18:0, and C18:2 *n*-6 (LA). SFA content decreased during the course of lactation (*p* = 0.0001, Table 3).

SCSFA, MCSFA, and LCSFA levels were highest in the 10th week of lactation. A statistically significant dependence was found between the levels of all groups of SFAs and the number of foalings. The milk of mares with ≤7 foalings had a higher saturated fatty acid content compared with milk produced by mares with >7 foalings (*p* = 0.0132). The SFA content decreased during lactation. A higher decrease was observed for lauric (C12:0, −35.94%) and myristic acids (C14:0, −22.63%) and lower decrease for palmitic acid (C 16:0, −9.06%). From a nutritional standpoint, C 18:0 has a neutral health effect and C 12:0, C 14:0, and C 16:0 have negative health effects [35]. The latter SFAs are considered dangerous because they are associated with high serum LDL-cholesterol concentrations in humans [35]. A similar dependence to that observed in our study was found in a study by Baretto et al. [31] on milk produced by Quarter Horse mares and in a study by Martemucci and D’Alessandro [15] on milk obtained from nine jennies of the Martina Franca breed, aged between six and 12 years. Our study found that both the stage of lactation and the number of foalings had a statistically significant effect on the levels of unsaturated fatty acids, which increased as lactation progressed and were higher in milk obtained from mares with >7 foalings. The contents of PUFAs were cca 10% lower than those of MUFAs. The contents of both fatty acid groups were significantly affected by the stage of lactation (*p* = 0.0001). The levels of those fatty acids were lowest in the 10th week of lactation. The levels of MUFAs in the milk of mares with >7 foalings were statistically significantly higher compared with milk obtained from the animals that had foaled a fewer number of times (*p* = 0.0008). However, no dependence was found between the number of foalings and PUFA levels (*p* = 0.0712). A study by Baretto et al. [31] on the milk of Quarter Horse mares found that UFA levels increased as lactation progressed, which was similar to our findings. In that study, the average concentration of UFAs in the period from the 121st to the 189th day of lactation was 52.21%. In their study on the milk of primitive Konik mares (*Equus caballus gmelini*), Pikul et al. [33] found a similar dependence of UFA levels in the 6th month of lactation, reaching 56.82%. These authors also found a statistically highly significant dependence between birth order (number of foalings) and UFA levels, which were higher in milk produced by mares with >5 foalings [33].

Among the PUFAs of the *n*-6 series presented in Table 3, the most abundant FA was linoleic acid (LA) (C18:2 *n*-6, 15.83% on average). LA levels were lowest in the 10th week (13.34%, *p* = 0.0001), with the highest LA content observed in the 15th and 25th week (16.33% and 17.55%, respectively).

The most represented PUFA of the *n*-3 series was alpha-linolenic acid, C18:3 *n*-3 (ALA)—5.26% on average. ALA levels were lowest in the 10th week—4.41%. In turn, the highest ALA content (6.19%) was observed in the 15th week (*p* = 0.0028). The PUFA *n*-6/*n*-3 ratio was 3.161 on average. The value of the parameter was statistically significantly dependent on the stage of lactation (*p* = 0.0163). The best PUFA *n*-6/*n*-3 ratio was observed in the 15th week post-partum. 

In our study, *n*-3 and *n*-6 PUFA levels were similar to those found in a study by Baretto et al. [31]. In that study, the PUFA *n*-6/*n*-3 ratio in milk obtained between the 61st and the 120th day of lactation was 3.062 [31], which was similar to the PUFA *n*-6/*n*-3 ratio found in our study.

For other minor PUFAs such as DLGA, (C20:3 *n*-6), arachidonic (AA, C20:4 *n*-6), eicosapentaenoic (EPA, C20:5 *n*-3), eicosatrienoic (C20:3 *n*-3), and decosapentaenoic (DPA, C22:5 *n*-3) acid, their average concentration was 0.094%, 0.096%, 0.117%, 0.173%, and 0.101%, respectively. Their concentration was not dependent on the stage of lactation, the number of foalings, and the age of the mares (*p* > 0.05).

With regard to the DFAs, their average concentration was 54.78% (Table 3), with a significant increase from week 10 and with a maximum concentration in the 25th week (*p* = 0.0001). Similar levels of desirable fatty acids (UFA + C18:0) to those observed in our study were found by Pikul and Wójtowski [20], Baretto et al. [31], and Navratilova et al. [36] in their studies on the milk of different breeds of mares.

The mean atherogenic index and the mean thrombogenic index were 1.059 and 0.678, respectively (Table 3). The atherogenic and thrombogenic indices were statistically significantly influenced by the stage of lactation (*p* = 0.0001). The atherogenic index was also statistically significantly influenced by birth order (*p* = 0.0366). The highest AI value was recorded in the 10th week of lactation. The AI decreased as lactation progressed, with the lowest and most health-beneficial AI value found in the 25th week of lactation. The 10th week of lactation was also the time when the highest thrombogenic index value was recorded. The TI values calculated in the 15th and 25th week of lactation were similar and were significantly lower than the value of the index in the first of the lactation periods analyzed (*p* = 0.0001). The AI and TI values recorded in our study were similar to the values of the indices found in a study by Pikul et al. [33] on the milk of primitive Konik mares (*Equus caballus gmelini*). The authors of the study also found a statistically highly significant dependence between birth order (number of foalings) and AI values, which were lower in milk derived from older mares [33]. In a study by Baretto et al. [31] on the milk of Quarter Horse mares, the AI in the periods from the 61st to the 120th day of lactation and from the 121st to the 180th day of lactation was 1.312 and 0.995, respectively, whereas the TI was 0.865 and 0.415, respectively.

## 4. Conclusions

The study found that the stage of lactation had a significant effect on the fatty acid profile of milk produced on the farm. The highest concentration of monounsaturated and polyunsaturated FAs was found in milk obtained from the 15th week of lactation. The fatty acid composition of milk obtained in that period is exceptionally beneficial for health. This was confirmed by the low values of the atherogenic and thrombogenic indices and the high concentration of desirable fatty acids. In light of these results, consideration may be given to postponing milking by a few weeks to benefit the development of foals and improve the health-promoting properties of consumer milk. One finding from the study that may be important for the management of farms is the significant effect of the number of foalings (birth order) on SFA, UFA, MUFA, and PFA levels and on atherogenic indices. These results indicate that it is possible to modify the fatty acid profile of milk by appropriately managing the age structure of the herd of mares. To confirm this dependence, the study will be continued on a larger group of mares.

## Figures and Tables

**Table 1:** Effect of lactation stage, birth order, and mares’ age on chemical composition and somatic cell count of mare’s milk.

Variable	$\bar{x}$	Lactation Stage in Week			No. of Foalings		SE	F	Lactation Stage	Birth Order	Mare’s Age
		10	15	25	≤7	>7			*p*-Value		
Fat [%]	1.16	1.14	1.11	1.22	1.21 ^c^	1.06 ^d^	0.04	2.45	0.3443	0.0474	0.0789
Protein [%]	2.00	2.25 ^a^	1.98 ^b^	1.84 ^b^	1.99	2.00	0.04	7.48	0.0001	0.0594	0.0491
Lactose [%]	6.44	6.33 ^a^	6.72 ^a^	6.19 ^b^	6.62 ^c^	6.11 ^d^	0.09	11.52	0.0139	0.0321	0.0338
Dry matter [%]	9.49	9.65 ^a^	9.58 ^a,b^	9.28 ^b^	9.50	9.48	0.06	2.54	0.0266	0.2957	0.2240
SCC * (10^3^/cm^3^)	16.29	9.13 ^a,b^	5.86 ^a^	33.25 ^b^	22.00	5.83	4.84	2.90	0.0279	0.3569	0.6938
LN (SCC) **	8.80	8.72	8.41	9.32	8.94	8.55	0.21	1.28	0.1560	0.3628	0.5882

* SCC—somatic cells count; ** LN (SCC)—logarithmic transformation of SCC; Means marked with different letters differ statistically (*p* ≤ 0.05): a, b—for lactation stage, c, d—for number of foalings.

**Table 2:** Effect of lactation stage, birth order, and mares’ age on fatty acid profile of mare’s milk (g/100 g total fatty acids).

FA	$\bar{x}$	Lactation Stage in Week			Birth Order		SE	F	Lactation Stage	Birth Order	Mare’s Age
		10	15	25	≤7	>7			*p*-Value		
C4:0	0.190	0.272	0.190	0.106	0.228	0.161	0.032	1.73	0.1470	0.1164	0.1903
C6:0	0.202	0.275 ^a^	0.194 ^b^	0.141 ^b^	0.213 ^d^	0.194 ^e^	0.013	7.95	0.0001	0.0175	0.0240
C8:0	2.516	3.022 ^a^	2.534 ^b^	1.982 ^c^	2.529 ^d^	2.506 ^e^	0.091	12.01	0.0001	0.0063	0.0051
C10:0	5.522	7.526 ^a^	5.198 ^b^	4.006 ^c^	5.771 ^d^	5.336 ^e^	0.253	41.73	0.0001	0.0001	0.0001
C10:1	1.436	1.596	1.433	1.281	1.333	1.513	0.053	2.20	0.0727	0.4954	0.9012
C12:0	6.769	8.733 ^a^	6.243 ^b^	5.595 ^b^	6.802	6.744	0.251	16.98	0.0001	0.1960	0.1446
C12:1	0.219	0.191	0.231	0.229	0.219	0.218	0.010	0.70	0.2674	0.9961	0.9409
C13:0	0.101	0.100	0.104	0.098	0.102	0.100	0.003	0.32	0.8377	0.4305	0.5078
C14:0	7.195	8.566 ^a^	6.659 ^b^	6.628 ^b^	7.429	7.019	0.218	6.74	0.0001	0.1506	0.2005
C14:1	0.746	0.720 ^a,b^	0.703 ^a^	0.835 ^b^	0.735	0.753	0.021	2.86	0.0450	0.0903	0.1236
C15:0	0.460	0.420	0.470	0.488	0.506	0.426	0.018	2.17	0.2657	0.2306	0.6392
C15:1	0.144	0.150	0.144	0.136	0.134	0.151	0.020	0.15	0.9437	0.6528	0.5412
C16:0	20.535	21.547 ^a^	19.595 ^b^	20.933 ^a,b^	20.920	20.246	0.289	2.72	0.0165	0.4502	0.5828
C16:1	6.207	5.721 ^a^	5.802 ^a^	7.302 ^b^	6.020 ^d^	6.347 ^e^	0.166	10.51	0.0001	0.0101	0.0315
C17:0	0.510	0.451 ^a^	0.519 ^a,b^	0.557 ^b^	0.513	0.508	0.016	1.79	0.0449	0.6082	0.6414
C17:1	0.419	0.247 ^a^	0.480 ^b^	0.497 ^b^	0.414	0.422	0.021	20.11	0.0001	0.7235	0.8084
C18:0	1.312	1.699 ^a^	1.119 ^b^	1.215 ^b^	1.297 ^d^	1.323 ^e^	0.053	19.43	0.0001	0.0040	0.0007
C18:1 *trans*	0.129	0.123	0.149	0.106	0.164	0.103	0.020	1.11	0.3989	0.7398	0.3679
C18:1 *cis-9*	21.428	18.309 ^a^	22.765 ^b^	22.542 ^b^	20.522	22.108	0.382	22.77	0.0001	0.3698	0.9744
C18:1 *cis-11*	1.146	1.005 ^a^	1.144 ^b^	1.290 ^c^	1.079	1.197	0.029	8.03	0.0001	0.9668	0.3964
C18:2 *n*-6 (LA)	15.826	13.337 ^a^	16.332 ^b^	17.558 ^b^	15.536 ^d^	16.045 ^e^	0.443	6.74	0.0001	0.0294	0.0328
C18:3 *n*-3 (ALA)	5.265	4.418 ^a^	6.198 ^b^	4.714 ^a^	5.877	4.806	0.310	4.46	0.0028	0.5229	0.1377
C18:2 *cis9 trans11* (CLA)	0.191	0.150 ^a^	0.181 ^a,b^	0.248 ^b^	0.222	0.168	0.014	3.72	0.0166	0.0814	0.2378
C20:0	0.096	0.098	0.095	0.097	0.099	0.094	0.004	0.14	0.9584	0.5403	0.6318
C20:1	0.403	0.363 ^a^	0.446 ^b^	0.377 ^a^	0.362 ^d^	0.434 ^e^	0.012	7.63	0.0017	0.0112	0.0908
C20:2	0.370	0.317 ^a^	0.386 ^b^	0.398 ^b^	0.342 ^d^	0.390 ^e^	0.012	3.16	0.0243	0.0429	0.9741
C20:3 *n*-6	0.094	0.091	0.097	0.091	0.093	0.094	0.004	0.20	0.8320	0.7070	0.6871
C20:4 *n*-6	0.096	0.091	0.101	0.093	0.094	0.097	0.004	0.55	0.8143	0.3596	0.3081
C20:5 *n*-3 EPA	0.117	0.154	0.102	0.102	0.095	0.133	0.019	0.72	0.4338	0.6285	0.5920
C20:3 *n*-3	0.173	0.140	0.199	0.158	0.200	0.156	0.014	1.60	0.2185	0.6183	0.9935
C22:5 *n*-3	0.101	0.097	0.104	0.102	0.098	0.104	0.004	0.22	0.8219	0.7641	0.9089

Means (in rows) marked with different letters differ statistically (*p* ≤ 0.05): a, b, c—for lactation stage, d, e—for number of foalings.

**Table 3:** Effect of lactation stage, birth order, and mares’ age on values of healthy fatty acid indices of mare’s milk (g/100 g total fatty acids).

FA Group/Index	$\bar{x}$	Lactation Stage in Week			Birth Order		SE	F	Lactation Period	Birth Order	Mare’s Age
		10	15	25	≤7	>7			*p*-Value		
SFA	45.409	52.709 ^a^	42.919 ^b^	41.844 ^b^	46.410 ^d^	44.658 ^e^	0.962	16.02	0.0001	0.0132	0.0185
SCSFA	0.392	0.547 ^a^	0.384 ^a,b^	0.248 ^b^	0.441 ^d^	0.355 ^e^	0.037	4.61	0.0040	0.0238	0.0477
MCSFA	14.807	19.281 ^a^	13.975 ^b^	11.582 ^c^	15.102 ^d^	14.586 ^e^	0.571	26.08	0.0001	0.0046	0.0044
LCSFA	30.210	32.880 ^a^	28.560 ^b^	30.014 ^b^	30.866	29.718	0.515	4.56	0.0011	0.1911	0.2568
MUFA	32.383	28.554 ^a^	33.397 ^b^	34.692 ^c^	31.097 ^d^	33.348 ^e^	0.447	66.89	0.0001	0.0008	0.0723
PUFA	22.207	18.737 ^a^	23.683 ^b^	23.464 ^b^	22.493	21.993	0.665	5.17	0.0005	0.0837	0.0381
UFA	54.591	47.291 ^a^	57.080 ^b^	58.156 ^b^	53.590 ^d^	55.341 ^e^	0.962	16.02	0.0001	0.0132	0.0185
PUFA_SFA	0.510	0.360 ^a^	0.569 ^b^	0.571 ^b^	0.510	0.510	0.025	6.49	0.0001	0.0712	0.0434
MUFA_SFA	0.733	0.544 ^a^	0.789 ^b^	0.838 ^b^	0.690 ^d^	0.765 ^e^	0.024	21.78	0.0001	0.0135	0.0519
LA_ALA	3.310	3.086 ^a^	3.027 ^a^	3.958 ^b^	2.846	3.658	0.169	4.36	0.0213	0.1516	0.6032
n6_PUFA	16.372	13.813 ^a^	16.899 ^b^	18.140 ^b^	16.034 ^d^	16.626 ^e^	0.454	6.74	0.0001	0.0295	0.0353
n3_PUFA	5.644	4.773 ^a^	6.603 ^b^	5.076 ^a,b^	6.237	5.199	0.319	4.29	0.0031	0.4843	0.1323
PUFA *n*-6/*n*-3	3.161	2.958 ^a^	2.901 ^a^	3.752 ^b^	2.760	3.461	0.150	4.38	0.0163	0.1923	0.6927
DFA	54.782	47.441 ^a^	57.261 ^b^	58.404 ^b^	53.812	55.509	0.965	16.08	0.0001	0.0147	0.0199
AI	1.059	1.380 ^a^	0.936 ^b^	0.922 ^b^	1.108 ^d^	1.022 ^e^	0.044	12.22	0.0001	0.0366	0.0502
TI	0.678	0.843 ^a^	0.594 ^b^	0.639 ^b^	0.693	0.667	0.027	6.81	0.0001	0.0839	0.0707

Means (in rows) marked with different letters differ statistically (*p* ≤ 0.05): a, b, c—for lactation stage, d, e—for number of foalings.

## Data Availability

The data presented in this study are available on request from the corresponding author.
